# Hazard Prevention, Death and Dignity During COVID-19 Pandemic in Italy

**DOI:** 10.3389/fpubh.2020.00509

**Published:** 2020-09-18

**Authors:** Silvia Ussai, Benedetta Armocida, Beatrice Formenti, Francesca Palestra, Marzia Calvi, Eduardo Missoni

**Affiliations:** ^1^Directorate General for Food and Health, European Commission, Brussels, Belgium; ^2^Institute for Maternal and Child Health, IRCCS “Burlo Garofolo”, Trieste, Italy; ^3^Saluteglobale.it Associazione di Promozione Sociale, Brescia, Italy; ^4^Center for Research on Health and Social Care Management (CERGAS), Bocconi University, Milan, Italy

**Keywords:** COVID-19, hazard & risk, deaths, public health, policy & institutional actions

## Abstract

On 9 March 2020, Italy passed the Prime Minister's Decree n. 648, establishing urgent measures to contain the transmission of COVID-19 and prevent biological hazards, including very restrictive interventions on public Holy Masses and funerals. Italy banned burial procedures based (i) on the recent acknowledgment about the virus environmental stability as well as (ii) its national civil contingency plan. Hence, only the cremation process is admitted for COVID-19 deaths. Viewing of the body is permitted only for mourners, which are allowed to perform the prayer at the closing of the coffin and the prayer at the tomb (cf. Rite of Succession, first part n. 3 and n. 5). The dead cannot be buried in their personal clothes; however, priests have been authorized to put the family clothes on top of the corpse, as if they were dressed. Burying personal items is also illegal. The dignity of the dead, their cultural and religious traditions, and their families should be always respected and protected. Among all the threats, COVID-19 epidemic in Italy revealed the fragility of human beings under enforced isolation and, for the first time, the painful deprivation of families to accompany their loved ones to the last farewell. Ethics poses new challenges in times of epidemics.

On March 24, 2020, the World Health Organization (WHO) released its guideline on “Infection Prevention and Control for the safe management of a dead body in the context of COVID-19” (1).

The document offers the most updated recommendations on the safe and dignified burial procedures of deceased persons with suspected or confirmed COVID-19. These include, among others: (1) the lack of evidence—which does not mean that we may exclude the possibility of future evidence emerging—of human transmission to subjects having become infected from exposure to the bodies of persons who died with/for COVID-19. This, in contrast with Ebola or Marburg diseases, where dead bodies are known to be associated with contagion; (2) the option for decedents with confirmed or suspected COVID-19 to be buried or cremated; (3) respect of customs, with family's chance to view the body after it has been prepared for burials, using standard precautions at all times including hand hygiene; (4) body wrapping in cloth and deceased transfer as soon as possible to the mortuary area. WHO recommendations are released in the form of interim guidance, subjected to revision as new evidence becomes available. National healthcare authorities are fully empowered in leading local actions according to the context and customs.

Italian Government adopted the highest level of precautions given its exceptional number of deaths (34.561, 20 June 2020) and the limited knowledge on this novel virus.

On 9 March 2020, Italy passed the Prime Minister's Decree n. 648 (2), establishing urgent measures to contain the transmission of COVID-19 and prevent biological hazards, including very restrictive interventions on public Holy Masses and funerals.

In coordination with the measures launched by the Italian authorities, the Italian Conference of Bishops (Conferenza Episcopale Italiana) issued a statement describing actions taken by the Vatican to limit the spread of COVID-19 (3). Severe measures that entail stringent restrictions on freedom of movement and association affecting the right to decent burials can be hugely distressing for families, exacerbating their grief. When balanced against public health interests, a basic rule is that governments should employ the least restrictive means necessary to protect public health.

During the emergency phase, Italy banned burial procedures based (i) on the recent acknowledgment about the virus environmental stability (4) as well as (ii) its national civil contingency plan. Hence, only the cremation process was admitted for COVID-19 deaths.

The visit to the body was equally forbidden by the Health Authority. Therefore, in addition to the funeral ceremonies, any prayer at the closing of the coffin was suspended as well.

Viewing of the body was permitted only for mourners, which were allowed to perform the prayer at the closing of the coffin and the prayer at the tomb (cf. Rite of Succession, first part n. 3 and n. 5).

The dead could not be buried in their personal clothes; however, priests have been authorized to put the family clothes on top of the corpse, as if they were dressed. Burying personal items was also illegal.

Funeral gatherings were not permitted and family members of SARS-CoV-2 victims were either denied to participate at the burial as they themselves were, most of time, under quarantine. In order to minimize delays between time of death and cremation, deceased were taken straight to the cemetery where a brief rite of burial was celebrated. All the Masses in suffrage of the deceased with the family have been postponed after the emergency.

Italy's mortuary industry has been overwhelmed as the number of dead kept rising. In Bergamo, a city in Lombardy region with the highest number of COVID-19 cases in Italy, the capacity to manage dead bodies exceeded. The time frame set by law from the death to the burial was up to 48 h. However, due to the unprecedented amount of deaths, certain areas experienced a 30 min turnover procedure because of the pressure created by the number of corpses, as caskets have been piling up in churches instead of the local cemeteries, which were full. The military stepped in to move about 70 coffins to other provinces and regions for timely burial procedures (5).

The cremation followed the standard procedure foreseen for biological hazard risk. For instance, in order to increase the capacity of each burial facility and in compliance with all hygiene requirements, safety and environmental regulations, alternative technical solutions have been allowed for each cremation to shorten the burial execution time, for example by accelerating the ignition of the coffin. The use of easily inflammable wooden coffins has been encouraged in cremation and only the use of a zinc inner bonnet was permitted.

In case of massive transportation of crematorium coffins, these have been carried out with a closed truck, also military, to be disinfected properly after use, preferably internally covered with waterproof material easily washable. Furthermore, in the cemetery register it was mandatory to indicate that the coffin was packed for the burial of the deceased with a contagious infectious disease by affixing the code “Y” (6).

Currently, the recovery phase is easing restrictions on funeral and burial procedures. Family members (up to 15 people) can participate in the Holy Mass; any physical contact between the participants must be avoided. The funeral should preferably take place outdoors and it is mandatory the use of Personal Protective Equipment (PPE) as well as the strict observance of the interpersonal safety distance of at least 1 m (7).

On March 31, 2020 the Italian government proclaimed a national day of mourning, inviting all public institutions to expose the Italian flag at half-mast “as a sign of mourning for the victims of the Coronavirus, of proximity to their families and of national participation in the condolences to the most affected communities.”

Authors acknowledge that in the absence of any pharmaceutical intervention, the only strategy against COVID-19 was to reduce mixing of susceptible and infectious people through early ascertainment of cases or reduction of contact. Although biologic hazard prevention actions (including death management) have been praised by WHO, the possibility of imposing severe restrictions on death during COVID-19 as adopted by the Italian government raises important questions. The population requires and deserves assurance that the decision to enact these measures affecting vital cultural practices as faith-based services has been informed by the best attainable evidence.

It is therefore relevant that policy makers maintain the public's trust through use of rigorous scientific assessment of risk and effectiveness.

Yet, burial restrictions in Italy have been imposed without any individualized risk assessment.

Difficult questions will then arise, though. For example, was complete funeral ban necessary, or might the final goodbye be still said by families while practicing physical distancing?

The dignity of the dead, their cultural and religious traditions, and their families should be always respected and protected. Among all the threats, COVID-19 epidemic in Italy revealed the fragility of human beings under enforced isolation and, for the first time, the painful deprivation of families to accompany their loved ones to the last farewell.

## Data Availability Statement

Publicly available datasets were analyzed in this study. This data can be found here: http://www.protezionecivile. gov.it.

## Author Contributions

All persons listed as authors have contributed to preparing the manuscript and their authorship meets the International Committee of Medical Journal Editors (ICMJE) criteria.

## Conflict of Interest

The authors declare that the research was conducted in the absence of any commercial or financial relationships that could be construed as a potential conflict of interest.
